# Dietary supplements and risk of cause-specific death, cardiovascular disease, and cancer: a protocol for a systematic review and network meta-analysis of primary prevention trials

**DOI:** 10.1186/s13643-015-0029-z

**Published:** 2015-03-26

**Authors:** Lukas Schwingshackl, Georg Hoffmann, Brian Buijsse, Tamara Mittag, Marta Stelmach-Mardas, Heiner Boeing, Marion Gottschald, Stefan Dietrich, Maria Arregui, Sofia Dias

**Affiliations:** Department of Nutritional Sciences, Faculty of Life Sciences, University of Vienna, Althanstraße 14 (UZA II),, 1090 Vienna, Austria; German Institute of Human Nutrition Potsdam-Rehbruecke (DIfE), Arthur-Scheunert-Allee 114-116, 14558 Nuthetal, Germany; Department of Gastroenterology and Metabolism, Poznan University of Medical Sciences, Poznan, Poland; School of Social and Community Medicine, University of Bristol, Canynge Hall 39, Whatley Road, BS8 2PS Bristol, UK

**Keywords:** Dietary supplements, Mortality, Incidence, Systematic review, Network meta-analysis, Cancer, Cardiovascular

## Abstract

**Background:**

In the Western world, dietary supplements are commonly used to prevent chronic diseases, mainly cardiovascular disease and cancer. However, there is inconsistent evidence on which dietary supplements actually lower risk of chronic disease, and some may even increase risk. We aim to evaluate the comparative safety and/or effectiveness of dietary supplements for the prevention of mortality (all-cause, cardiovascular, and cancer) and cardiovascular and cancer incidence in primary prevention trials.

**Methods/Design:**

We will search PubMed, EMBASE, Cochrane Database of Systematic Reviews, the Database of Abstracts of Reviews of Effects, the Cochrane Central Register of Controlled Trials, clinical trials.gov, and the World Health Organization International Trial Registry Platform. Randomized controlled trials will be included if they meet the following criteria: (1) minimum intervention period of 12 months; (2) primary prevention of chronic disease (is concerned with preventing the onset of diseases and conditions); (3) minimum mean age ≥18 years (maximum mean age 70 years); (4) intervention(s) include vitamins (beta-carotene, vitamin A, B vitamins, Vitamin C, Vitamin D, Vitamin E, and multivitamin supplements); fatty acids (omega-3 fatty acids, omega-6 fatty acids, monounsaturated fat); minerals (magnesium, calcium, selenium, potassium, iron, zinc, copper, iodine; multiminerals); supplements containing combinations of both vitamins and minerals; protein (amino acids); fiber; prebiotics; probiotics; synbiotics; (5) supplements are orally administered as liquids, pills, capsules, tablets, drops, ampoules, or powder; (6) report results on all-cause mortality (primary outcome) and/or mortality from cardiovascular disease or cancer, cardiovascular and/or cancer incidence (secondary outcomes).

Pooled effects across studies will be calculated using Bayesian random effects network meta-analysis. Sensitivity analysis will be performed for trials lasting ≥5 years, trials with low risk of bias, trials in elderly people (≥65 years), ethnicity, geographical region, and trials in men and women. The results of the corresponding fixed effects models will also be compared in sensitivity analyses.

**Discussion:**

This is a presentation of the study protocol only. Results and conclusions are pending completion of this study. Our systematic review will be of great value to consumers of supplements, healthcare providers, and policy-makers, regarding the use of dietary supplements.

**Systematic review registration:**

PROSPERO: CRD42014014801.

**Electronic supplementary material:**

The online version of this article (doi:10.1186/s13643-015-0029-z) contains supplementary material, which is available to authorized users.

## Background

The use of dietary supplements has increased over time in the United States. In the National Health and Nutrition Examination Survey I, the prevalence of dietary supplement use was 28% among males and 38% among females. The most recent data indicate that about one-half of the U.S. population and 70% of adults ≥71 years of age use dietary supplements. The most predominant supplements are multivitamin-multi-mineral supplements, which account for about one third [1]. Magnesium was the most used mineral dietary supplement [1]. High quality data for comparisons among European countries are sparse [2]. The largest European cohort study until to date, the European Prospective Investigation into Cancer and Nutrition study, indicates that there are significant differences in dietary supplement intake in Europe, varying between 2% in Greek men and 66% in Danish women [3]. Overall dietary supplement use was more prevalent in Northern European countries compared to southern countries, especially higher intakes of cod liver oil could be observed [3].

There is evidence that specific populations such as cancer survivors have a higher intake of dietary supplements, compared to the general healthy population [4,5].

Previous pairwise meta-analyses of randomized controlled trials showed inconsistent effects of dietary supplements [6,7]. One of the largest meta-analysis showed that treatment with beta-carotene, vitamin A, and vitamin E may increase mortality [8], whereas vitamin D supplementation seems to be associated with reduced mortality [9,10]. Vitamin B supplementation has a significant protective effect on stroke, but no effect on CVD mortality or cancer [11,12], whereas calcium supplementation has no significant effects on cancer risk [13]. Omega-3 fatty acids are probably the most studied dietary supplements, and recent meta-analyses showed no protective effects on cardiovascular disease [14,15]. However, there seems to be some differences between primary and secondary prevention trials, between lower *vs.* higher dose of omega-3 fatty acids, and between different clinical endpoints [16,17].

To date, no systematic review and meta-analysis has jointly synthesized the direct and indirect evidence of the effects of all dietary supplements on all-cause mortality, cardiovascular mortality, cancer mortality, incidence of CVD, and cancer. Therefore, we aim to summarize all the available evidence on dietary supplements and mortality (all-cause, cardiovascular, cancer) and incidence (cardiovascular disease, cancer) as well as to assess the efficacy and safety of different dietary supplements in primary prevention trials.

## Methods/Design

The review was registered in PROSPERO International Prospective Register of Systematic Reviews (www.crd.york.ac.uk/prospero/index.asp, identifier CRD42014014801). The present systematic review protocol was planned, conducted, and reported in adherence to standards of quality for reporting systematic review and meta-analysis protocols (PRISMA-P) [18,19].

### Eligibility criteria

Studies will be included in the meta-analysis if they meet all of the following criteria:

1. Randomized controlled design (identical placebo or no intervention) or trials of one supplement *vs*. another;
2. Minimum intervention period of 12 months;
3. Primary prevention (of chronic disease) trials (trials concerned with preventing the onset of diseases and conditions);
4. Minimum mean age ≥18 years;
5. Intervention: *dietary supplements defined according to the Directive 2002/46/EC of the European parliament and of the Council, of 10 June 2002* [20]; the following dietary supplements will be included (according to previous systematic reviews and meta-analyses on dietary supplements and chronic diseases [8,10,15]); vitamins (beta-carotene, Vitamin A, B vitamins (thiamin, riboflavin, niacin, pyridoxine, cobalamin, folic acid), Vitamin C (ascorbic acid), Vitamin D (cholecalciferol, ergocalciferol, alfacalcidol, calcitriol), Vitamin E, and multivitamin supplements) supplements containing a combination of different vitamins; fatty acids: omega-3 fatty acids (eicosapentaenoic acid, docosahexaenoic acid, α-linolenic acid); omega-6 fatty acids (linoleic acid); monounsaturated fat (olive oil); minerals: magnesium, calcium, selenium, potassium, iron, zinc, copper, iodine; multi-minerals; and supplements containing combinations of both vitamins and minerals; protein (amino acids: alanine, arginine, asparagine, aspartic acid, cysteine, glutamic acid, glutamine, glycine, proline, selenocysteine, serine, tyrosine, isoleucine, leucine, lysine, methionine, phenylalanine, threonine, tryptophan, valine); fiber (psyllium, inulin, cellulose); probiotics (‘viable microorganisms, sufficient amounts of which reach the intestine in an active state and thus exert positive health effects’: *Lactobacillus rhamnosus GG, Lactobacillus reuteri, bifidobacteria* and certain strains of *Lactobacillus casei* or the *Lactobacillus acidophilus-group*, *Escherichia coli strain Nissle 1917, certain enterococci* (*Enterococcus faecium SF68*) and the probiotic yeast *Saccharomyces boulardii*); prebiotics (‘a selectively fermented ingredient that allows specific changes, both in the composition and/or activity in the gastrointestinal microflora that confers benefits upon host well being and health’: oligofructose and (trans)galactooligosaccharides); synbiotics (‘synergistic combinations of pro- and prebiotics’) [21];
6. Oral intake: modalities of supplement intake: liquid, pill, capsule, tablet, drops, ampoule, powdered;
7. Assessment of the ‘primary’ outcomes: all-cause mortality, ‘secondary’ outcomes: cardiovascular mortality, cancer mortality; cardiovascular incidence, and cancer incidence (trials must report at least one of these outcomes).
8. Report the number of events, sample size, and follow-up time for each group, or report the hazard ratio with a measure of uncertainty or where there are sufficient details for this to be calculated (for example, from a confidence interval or *P* value).

### Exclusion criteria

1. Exclusion of studies with a dietary co-intervention that was not applied in all the intervention or placebo/control groups;
2. Exclusion of studies with a drug-intervention that was not applied in all the intervention or placebo/control groups (that is, trials allowing concomitant medications will be included if applied in all groups in a comparable regimen);
3. Studies with intravenous or parenteral administration of vitamins or minerals will be excluded;
4. Pregnant or lactating women will be excluded.
5. Mean age ≥70 years;
6. Non-primary prevention trials (>75% of sample size) will be excluded (defined as trials undertaken to prevent recurrences or exacerbations of a disease that has already been diagnosed: cancer survivors, survivor of myocardial infarction, stable/unstable angina pectoris, acute coronary insufficiency, coronary artery disease (verified by coronary angiography), stroke, hemodialysis, chronic kidney disease, and subjects with the following diseases: gastrointestinal, neurological, ocular, dermatological, rheumatoid, endocrinological).
7. Follow-up time is not reported.

### Study type

Only RCTs that are peer-reviewed and available in full-text are eligible for the present network meta-analysis. The following type of studies will be excluded: observational studies, case series, and case reports.

### Search strategy

We will conduct searches in Cochrane Central Register of Controlled Trials (CENTRAL) on the Cochrane Library, PubMed (from 1966), EMBASE (from 1980). A highly sensitive RCT filter will be used with the PubMed search, as recommended by the Cochrane Handbook (‘randomized controlled trial’ OR ‘randomized’ OR ‘clinical trials as topic’ OR ‘placebo’ OR ‘randomly’ OR ‘trial’) NOT (‘animals’) [22]. We will also conduct searches in Clinicaltrials.gov (http://clinicaltrials.gov/) and the World Health Organization International Clinical Trials Registry Platform to look for ongoing trials. A comprehensive search strategy will be performed for unpublished data (contact with manufactures, FDA website, and request of study reports).

We will search for articles of original research by using the following search terms (Additional file 1). Moreover, the reference lists from the retrieved articles; systematic reviews and meta-analyses will be checked to search for further relevant studies. There will be no restrictions on language or publication year.

### Study selection process

Two reviewers will independently screen titles and abstracts of all the retrieved bibliographic records. Full texts of all potentially eligible records passing the title and abstract screening level will be retrieved and examined independently by two reviewers (for each database) with the above mentioned eligibility criteria/exclusion criteria [23,24]. Disagreements will be resolved by consensus or adjudication of another reviewer. A flow diagram will outline the study selection process and reasons for exclusions (full-text).

### Data extraction

After determination of the study selection, the following eligibility criteria will be extracted: first author’s last name, publication year, country of origin, study design, study duration, follow-up, study population, number of arms, participants’ sex and age, sample size, dietary supplement, dose (g/day), mode of administration, baseline risks (smoking, BMI, hypercholesterolemia, glycemia, blood pressure, co-medications), indication, specification of the control group, number of events (all-cause mortality, cardiovascular mortality, cancer mortality, cardiovascular incidence, cancer incidence) and hazard ratios, where reported, withdrawals and drop-outs, adverse events, and funding source. These variables will be extracted for all studies, after which the extracted data will be verified by a second reviewer to reduce reviewer errors and bias.

### Risk of bias assessment

Full copies of the studies will be independently assessed by two authors for methodological quality using the risk of bias assessment tool from the Cochrane Collaboration [22,25]. The following sources of bias will be detected: selection bias (random sequence generation and allocation concealment), detection bias (blinding of outcome assessment), blinding of participants and personnel (performance bias), attrition bias (incomplete outcome data), reporting bias (selective reporting), and industry bias.

### Quality of the evidence

The quality of the evidence will be rated according to the GRADE guidelines [26,27].

### Dealing with missing data

We will try to obtain relevant missing data from authors of the included trials (by mail).

### Statistical analysis

For each outcome measure of interest, pairwise and network random effects meta-analyses will be performed in order to determine the pooled relative effect of each intervention relative to every other intervention in terms of the hazard ratio of the intervention *vs*. control/placebo groups. In pairwise meta-analyses, heterogeneity between trial results will be tested with a Cochran’s Q test with a value for *I*^2^ of >50% considered to represent substantial heterogeneity [28]. Forest plots will be generated to illustrate the study-specific effect sizes along with a 95% CI. To determine the presence of publication bias, the symmetry of the funnel plots in which mean hazard ratios will be plotted against their corresponding standard errors for each comparison where the number of included trials is 10 or more. Additionally, Begg’s and Egger’s regression tests will be performed to detect small study effects [29,30]. Separate pairwise meta-analyses will be used to compare all the interventions first. Network meta-analysis will then be used to synthesize all the available evidence [31]. Network meta-analysis methods are extensions of the standard pairwise meta-analysis model that enable a simultaneous comparison of multiple interventions while preserving the internal randomization of individual trials. They have the advantage of adequately accounting for the correlation in relative effect estimates from multi-arm trials (trial with more than two arms) as well as providing a single coherent summary of all the evidence. Random effects network meta-analysis models will be used when substantial heterogeneity is found in any of the pairwise comparisons for that outcome. Otherwise, the choice between fixed and random effects will be made by comparing the deviance information criteria for each model [31,32]. The model with the lowest deviance information criterion will be preferred (differences >3 are considered meaningful). Pooled effect sizes from the network meta-analyses will be presented as posterior medians and 95% credible intervals (that is, the Bayesian equivalent of CIs) in the appropriate units, along with the estimated between-study heterogeneity and its 95% credible interval.

Placebo and no treatment will be considered as separate interventions. For supplements, different modalities of intake (liquid, pill, and so on) will also be considered separately. However, if the number of trials comparing different intake modalities is small, we will explore models that combine different modalities of intake of a supplement as a single treatment and will consider placebo and no treatment as equivalent. Such models will be acceptable if they are a good fit to the data and have small between-study heterogeneity. We do not expect there to be differences in non-active interventions (placebos) according to their intake modality. However, this will be explored in the NMA if there are enough data and we find substantial heterogeneity or inconsistency.

As trial follow-up times are expected to differ and more events are expected for longer follow-up times, all meta-analyses (pairwise and network) will be conducted on the log-hazard ratio scale. Hazard rates will be estimated taking into account trial follow-up for event data and incorporating hazard ratio data, when these are reported, using a shared parameter model [31,33]. Data from studies reporting the number of events at a given follow-up time will be modeled using complementary log-log regression, and hazard ratios with their uncertainty will be combined in the same NMA using a ‘shared parameter model’ [31,33]. Where studies report both the number of events and follow-up time and hazard ratios (with a measure of uncertainty), the latter will be preferred as this accounts for censoring.

For pairwise meta-analyses, data will be analyzed using Review Manager 5.1 software, provided by the Cochrane Collaboration (http://ims.Cochrane.org/revman) using the generic inverse variance method. Network meta-analyses will be conducted using Markov chain Monte Carlo simulation implemented with the open-source software WinBUGS, version 1.4.3 [34]. The WinBUGS code used is freely available online [31,33].

Minimally informative normal priors will be used for all treatment effect variables. Uniform priors will be used for the between-study standard deviation (heterogeneity).

Three Markov chain Monte Carlo chains will be used to assess convergence using Brooks-Gelman-Rubin plots and inspection of the trace plots [35]. Posterior summaries will then be obtained from further iterations in each of the three chains, with a sufficient number of iterations so that the resulting Monte Carlo error is small.

The potential for inconsistency will be assessed by inspection of the available evidence. In case of possible inconsistency, Bayesian *P* values for the difference between direct and indirect evidence will be calculated using the node-split method, and direct and indirect estimates will be compared [36,37].

We plan to perform sensitivity analyses for long-term intervention trials (≥5 years), low risk of bias trials and elderly people (≥65 years), and trials in men and women. Furthermore, it is planned (if the number of trials is high enough) to perform sensitivity analysis with respect to ethnicity and geographical region. The results of the corresponding fixed effects models will be also compared in sensitivity analyses.

## Discussion

This systematic review and network meta-analysis will be the first to pool and compare the effects of different dietary supplements on all-cause mortality, cardiovascular and cancer mortality, and incidence, using both direct and indirect evidence. Since dietary supplements are often used by populations in the Western world, it is important to detect the potential benefits and/or harms on hard clinical outcome parameters. Furthermore, this analysis will show which dietary supplements, if any, are the most efficacious in the prevention of the hard clinical outcome parameters or cause the greatest harm. Results and conclusions are pending completions of this study. Our network meta-analysis will be of utility to consumers of dietary supplements, healthcare providers, and policy-makers, regarding the use of dietary supplements.

## Additional file

Additional file 1:
**PubMed search strategy.**

## Abbreviations

CVD: cardiovascular disease
NMA: network meta-analysis
RCTs: randomized controlled trials
