# Supplementary material for: Dietary supplements and risk of cause-specific death, cardiovascular disease, and cancer: a protocol for a systematic review and network meta-analysis of primary prevention trials
Source: Syst Rev. 2015 Mar 26;4:34. doi: 10.1186/s13643-015-0029-z (PMC4379718; doi:10.1186/s13643-015-0029-z)
Supplement: Additional file 1: — PubMed search strategy. [file 13643_2015_29_MOESM1_ESM.docx]

**Additional file 1 PubMed search strategy.**

("supplements"[All Fields] OR "supplementation"[All Fields] OR "vitamins"[All Fields] OR "vitamin"[All Fields] OR "multivitamins"[All Fields] OR "multimineral"[All Fields] OR "cholecalciferol"[All Fields] OR "ergocalciferol"[All Fields] OR "alfacalcidol"[All Fields] OR "calcitriol"[All Fields] OR "ascorbic acid"[All Fields] OR "antioxidant"[All Fields] OR "antioxidants"[All Fields] OR "protein"[All Fields] OR "amino acids"[All Fields] OR "micronutrient"[All Fields] OR "micronutrients"[All Fields] OR "calcium"[All Fields] OR "magnesium"[All Fields] OR "potassium"[All Fields] OR "selenium"[All Fields] OR "iron"[All Fields] OR "zinc"[All Fields] OR "omega 3"[All Fields] OR "fatty acids"[All Fields] OR "fiber"[All Fields] OR "beta carotene"[All Fields] OR "folic acid"[All Fields] OR "niacin"[All Fields] OR "thiamine"[All Fields] OR "riboflavin"[All Fields] OR "eicosapentaenoic acid"[All Fields] OR "docosahexaenoic acid"[All Fields] OR "linolenic acid"[All Fields] OR "olive oil"[All Fields] OR "inulin"[All Fields] OR "psyllium"[All Fields] OR "cellulose"[All Fields] OR "copper"[All Fields] OR "iodine"[All Fields] OR "prebiotics"[All Fields] OR "probiotics"[All Fields] OR "synbiotics"[All Fields]) AND ("cardiovascular"[All Fields] OR "coronary"[All Fields] OR "cancer"[All Fields] OR "neoplasm"[All Fields] OR "neoplastic disease"[All Fields] OR "stroke"[All Fields]) AND ("mortality"[All Fields] OR "death"[All Fields] OR "incidence"[All Fields] OR "morbidity"[All Fields]) AND ("randomized controlled trial"[All Fields] OR "randomized"[All Fields] OR "clinical trials as topic"[All Fields] OR "placebo"[All Fields] OR "randomly"[All Fields] OR "trial"[All Fields]) NOT "animals"[All Fields]
